# Effects of the Affordable Care Act on the Receipt of Colonoscopies among the Insured Elderly

**DOI:** 10.3390/ijerph17010313

**Published:** 2020-01-02

**Authors:** Minjee Lee, M. Mahmud Khan, Heather M. Brandt, Ramzi G. Salloum, Brian Chen

**Affiliations:** 1 Department of Population Science and Policy, Southern Illinois University School of Medicine, 201 E. Madison St Room 106, Springfield, IL 62794, USA; 2 Department of Health Services Policy and Management, Arnold School of Public Health, University of South Carolina, 915 Greene St, Suite 357, Columbia, SC 29208, USA; mkhan@mailbox.sc.edu (M.M.K.); bchen@mailbox.sc.edu (B.C.); 3 Department of Health Promotion, Education, and Behavior, Arnold School of Public Health, University of South Carolina, 915 Greene St, Suite 546, Columbia, SC 29208, USA; hbrandt@sc.edu; 4 Department of Health Outcomes & Biomedical Informatics, University of Florida, College of Medicine, 2004 Mowry Road, Suite 2243, Gainesville, FL 32610, USA; rsalloum@ufl.edu

**Keywords:** colonoscopy, disparity, Affordable Care Act

## Abstract

Background: The Affordable Care Act (ACA) waived deductibles and eliminated coinsurance for colonoscopies for Medicare beneficiaries beginning in January 1, 2011. This study investigated the effect of the ACA’s directive to remove the financial barriers on the receipt of colonoscopies among the elderly insured, who are predominantly covered by Medicare. Methods: Data from the 2008–2016 Behavioral Risk Factor Surveillance System (BRFSS) were used to examine the receipt of colonoscopies in two years prior to the implementation of the ACA (2008 and 2010) and three years after the change (2012, 2014, and 2016). Multivariate logistic regressions were estimated to examine the change in colonoscopy use before and after the introduction of the ACA, adjusting for patient characteristics and availability of health care providers in the geographic region. Results: Of 349,899 eligible elderly insured in the age group 65 to 75 years, 236,275 (67.2%) had received a colonoscopy in the previous ten years. The receipt of colonoscopies increased from 63.5% in pre-ACA years to 69.2% in the post-ACA years (*p* < 0.001). Compared with the pre-ACA period, the odds ratio of colonoscopy uptake in post-ACA years was 1.15 (95% CI = 1.08–1.22). Conclusions: A statistically significant increase in colonoscopy use was observed in the post-ACA years. However, achieving the target coverage rate of 80% will require additional interventions to encourage higher levels of screenings.

## 1. Introduction

Colorectal cancer (CRC) is the second leading cause of cancer deaths for both men and women in the United States [1]. There were an estimated 135,430 new cases of and 50,260 deaths from CRC in 2017 [1]. Early detection of CRC through routine screening has been demonstrated to be effective in reducing the incidence of and mortality from this disease [1,2,3]. The U.S. Preventive Services Task Force (USPSTF) strongly recommends screening for CRC beginning at the age of 50 years and continuing until the age of 75 years for individuals at average risk. Mortality from CRC can be reduced significantly through increased screening for CRC [2,4,5,6]. 

The USPSTF recommends a number of screening tests for detecting early-stage CRC and preventing incident cases, including (1) flexible sigmoidoscopy every five years, (2) FIT-DNA every one or three years, (3) fecal occult blood test or fecal immunochemical testing every year, (4) CT colonography every five years, (5) flexible sigmoidoscopy every ten years plus FIT every year, and (6) colonoscopy every ten years [4]. The USPSTF recommends screening using any of the accepted methods, as any type of screening test would be better than no screening at all [4,7,8]. Colonoscopy allows doctors to examine the entire length of the colon and remove all cancers and precancerous polyps during a single procedure [9,10]. Colonoscopy is also recommended as a follow-up when another CRC screening is positive. 

The mortality rate from CRC has decreased steadily since 1980 [1,11], which may partially be attributable to the removal of pre-cancerous, adenomatous polyps at an early stage during colonoscopies [12,13]. Nonetheless, the colorectal cancer screening rate is quite low and significant improvements in CRC mortality can be achieved by increasing the coverage of CRC screenings [14,15]. The CRC screening rate is lower than the use of preventive interventions for other screening-amenable cancers and remained below the HP2020 target of 71% [16,17].

One potential barrier to CRC screening is the out-of-pocket financial costs associated with screening [18,19,20]. The financial costs may significantly dampen patients’ willingness to adopt any preventive procedure, including any type of CRC screening. This is especially true for colonoscopies, which usually involved a relatively high cost-sharing requirement prior to the Affordable Care Act (ACA) policy changes in 2011 [21,22]. Previous studies have shown that cost-sharing reduces preventive health care use, including the use of highly effective screening tests [23,24]. For example, one study found that waiving coinsurance for colonoscopies resulted in an 18% increase in screening [25].

To address the negative consequences of financial barriers on the use of preventive services and to promote CRC screening, the ACA required all non-grandfathered private health plans to offer coverage of CRC screening without cost-sharing. Consistent with the ACA policy requirement, beginning 1 January 2011, Medicare waived Part B deductibles for all colonoscopies and eliminated coinsurance for screening colonoscopies, though not for diagnostic ones [18,22]. Therefore, Medicare beneficiaries may face unexpected out-of-pocket liabilities when a polyp is detected and removed during a colonoscopy, as these patients are billed a copay. Medicare beneficiaries are also responsible for Part B deductibles and coinsurance when a colonoscopy is performed as part of a two-step screening process after another CRC screening is positive [18]. Nevertheless, the ACA policy change, in general, implies that the elderly insured population should see significant reductions in out-of-pocket expenses associated with colonoscopies. 

Research on the effects of cost-sharing reductions on the utilization of preventive health care has received significant attention from researchers and policy-makers, but surprisingly, only a few studies have assessed the effect of cost-sharing reduction on colonoscopies among the elderly insured population following the implementation of the ACA [22,26]. The few studies that have examined this issue have used a very short time frame beginning with the implementation of the ACA, so they may have underestimated the effects of the ACA cost-sharing reduction. Furthermore, the results of these studies vary regarding the receipt of colonoscopies following the changes in coverage post-ACA, [22,24,26] and they have not been able to determine whether eliminating financial barriers might have helped socioeconomically vulnerable Medicare beneficiaries more than other groups. 

To address these gaps in current research, this study aimed to examine the changes in colonoscopy use among the elderly insured population, including Medicare beneficiaries, following the implementation of the ACA policy for preventive services. 

## 2. Materials and Methods

### 2.1. Study Population

This study used 2008–2016 Behavioral Risk Factor Surveillance System (BRFSS) data, an annual, nationally representative survey of the United States population. BRFSS uses random-digit telephone dialing methods to sample noninstitutionalized adults aged 18 years or older [27]. In 2008, the BRFSS began including questions about colonoscopies in even years. Therefore, this study used data from the years 2008, 2010, 2012, 2014, and 2016. 

The sample for this study consists of noninstitutionalized, insured elderly aged 65 to 75 years who participated in the survey. For our analyses, only those insured who were in the age group 65 to 75 years were included, bringing the sample size down to 446,981 adults. We excluded individuals with missing values for variables of interest and those who refused to answer questions relevant to creating the main measures for the study. Thus, the analytic sample consisted of 349,899 participants aged 65 to 75 years. This study did not require ethical approval since we used publicly available datasets. 

### 2.2. Measures

The outcome of interest in our study is the self-reported receipt of colonoscopies in the previous 10 years. We defined our outcome variable as a dichotomous measure of whether an individual was up-to-date with the USPSTF screening recommendation. 

Based on previous studies [22,26,28], our analysis included demographic variables (age, sex, race and ethnicity, marital status, and region of residence) as possible covariates explaining the adoption of a colonoscopy. We incorporated race and ethnicity variables using the following discrete categories: non-Hispanic white, non-Hispanic Black, Hispanic, and other. Marital status was classified into two categories: married and other (divorced, widowed, separated, never married, and unmarried couple). Region of residence was classified into four census regions of the country based on FIPS codes: Northeast, Midwest, South, and West. 

We included two socioeconomic variables in the model as well (household income and educational attainment). Household income was reported using the following income classes: lower than $15,000; $15,000 to $25,000; $25,000 to $35,000; $35,000 to $50,000; and higher than $50,000. Educational attainment was grouped into four categories: did not graduate high school; graduated high school; attended college and; graduated from college.

We pooled all five cycles of the survey into one large data set. The data set includes individual surveys conducted in the two years prior to the ACA policy change (2008 and 2010) and the three years after (2012, 2014, and 2016). To capture the effect of policy change on the receipt of colonoscopies, a policy-shift dummy variable was introduced into the model (pre- and post-ACA years). 

We used the geographic availability of gastroenterologists and the degree of health awareness of surveyed individuals as possible covariates affecting the receipt of colonoscopies. The American Medical Association (AMA) Health Workforce Mapper (https://www.ama-assn.org/about/research/health-workforce-mapper) reports the availability of different specialists by state, and we used the reported number of professionally active gastroenterologists (GI) in the state to calculate geographic availability of GIs per 1000 individuals in a given population. We reported the geographic availability of gastroenterologists as a quartile of gastroenterologist availability, i.e., we divided the distribution of the variable across states into four equal groups. Previous studies have demonstrated that a greater provider supply has been associated with the increased use of colonoscopies [28,29]. 

The BRFSS does not have any direct measure of an individual’s awareness of colonoscopy as a screening option or the importance assigned by individuals to preventive services that have little or no current benefits but improved future health. One of the concerns in estimating the effect of a policy change over the years is that individuals may become more aware of the importance of colonoscopies as well as other preventive services. Over the years, the awareness level may improve due to ongoing campaigns. If we assume that knowledge about all the preventive services is interrelated, “effective” use of one or more of the preventive actions will also imply improvements in knowledge about the importance of colonoscopies. We decided to use two proxy measures for this purpose: participation in physical exercise and smoking status. The adoption of physical exercise reflects an individual’s willingness to accept a preventive activity to improve health in the future. Smokers are likely to discount future years at a much higher rate than non-smokers and former smokers. Those with a lower time preference rate (lower discount rate) are more likely to adopt preventive interventions and screenings [30,31]. 

### 2.3. Statistical Analysis

Descriptive statistics were used to summarize participant characteristics and to report the number and percentage of participants for each of the variables. We also reported the percentage of respondents who had a colonoscopy in the previous 10 years by pre- and post-ACA policy change. Bivariate and multivariate logistic models were used to estimate the effects of the policy-shift on the receipt of colonoscopies among insured elderly aged 65 to 75 years. The multivariate models adjusted the outcome variable for demographic characteristics, socioeconomic status, geographic availability of gastroenterologists, health awareness proxies, and the policy change shifter variable. Sampling weights were used to derive national estimates. All statistical analyses were performed using SAS version 9.4 (SAS Inc., Cary, NC, USA). Statistical significance was set at *p* < 0.05 or CI not including 1 and all tests were two-tailed. 

## 3. Results

Table 1 shows the descriptive statistics for pre-ACA years and post-ACA years in the total sample. The data set had 349,899 adults aged 65 to 75 years (Table 1). The majority of participants were female (51.6%), married (62.9%), had exercised in the last 30 days (72.2%), and had received a colonoscopy within the last ten years (67.2%).

Table 2 showed colonoscopy use before and after the implementation of the ACA. Overall, the receipt of colonoscopies increased from 63.5% in pre-ACA years to 69.2% in post-ACA years (*p* < 0.0001). Rates of colonoscopy use by household income and individual educational attainment indicated larger gains among the socioeconomically vulnerable elderly (Table 2).

Table 3 reports the results of the multivariate analysis of factors associated with colonoscopy use over the previous ten years. Increased use of colonoscopy was associated with older age, being female, exercise status, and smoking status. After controlling for demographic characteristics, socioeconomic status and other relevant variables, the policy shift variable was statistically significant, implying that colonoscopy use increased among the elderly insured after the implementation of the ACA (OR, 1.15; 95% confidence limit [CI], 1.08–1.22), given various socioeconomic, demographic and other relevant covariates. 

Individuals with a household income greater than $50,000 were 2.10 times more likely to have received a recommended colonoscopy compared with individuals whose household income was less than $15,000. Individuals who graduated from college were 1.53 times more likely to have received a recommended colonoscopy compared with individuals who did not graduate high school. We tested interaction terms combining the policy shift variable with age, sex, race/ethnicity, marital status, region of residence, educational attainment, household income, gastroenterologist availability, exercise, and smoking status; but none of the interaction terms were statistically significant. 

## 4. Discussion

Analysis of BRFSS data indicates that the receipt of colonoscopies among the elderly insured increased from 63.5% in pre-ACA years to 69.2% in post-ACA years. Elderly insured in the age group 65 to 75 years are 1.15 times more likely to be up-to-date with colonoscopy screening after the policy change compared to pre-ACA status, after controlling for a number of individual and geographic factors. Although the analysis could not incorporate out-of-pocket expenses directly into the model due to lack of data, it is likely that the increase in colonoscopy uptake observed in the post-ACA years was due to the reduction in cost-sharing. 

Consistent with earlier research findings [22], our results confirmed that there was a statistically significant increase in colonoscopy use among elderly beneficiaries aged 65 to 75 years after the implementation of the ACA. The results also correspond with prior literature showing a positive association between cost-sharing reduction and utilization of recommended preventive services [26,32,33,34]. However, even after a significant reduction in out-of-pocket expenses for receiving colonoscopies, the coverage of colonoscopy remains suboptimal and much lower than the 80% target by 2018. It is important to identify specific approaches to encourage socioeconomically disadvantaged elderly to seek colonoscopies in order to achieve a higher rate of progress in achieving the target, even though increases in colonoscopy uptake were the largest among the lower-income and education groups in the post-ACA years compared to the pre-ACA years.

Although the increase in coverage has been slow, we found a statistically significant increase in colonoscopy uptake among elderly insured with lower socioeconomic status after the implementation of the ACA. This may, in part, reflect the effect of removal of out-of-pocket costs, since financial barriers are found to reduce coverage of cancer screening [35], and colonoscopies are expensive [36]. It is clear that the increase was universal across socioeconomic status and not limited to subjects with lower income and lower levels of education. However, despite the improvements in colonoscopy uptake over the years, the poorest and the most socially disadvantaged groups represent the highest potential for improvement, given their relatively low rates of colonoscopy use. For achieving the target screening rate, additional interventions should be considered in addition to the lowering of out-of-pocket expenses. The ACA’s reduction of financial barriers has improved adherence to CRC screening, but other non-medical costs should be considered more carefully to rapidly improve the screening rates. 

There are several barriers to the receipt of colonoscopies other than the out-of-pocket cost, including perceived loss of utility associated with bowel preparation prior to the test, logistical challenges, not receiving a physician’s recommendation for CRC screening, and believing that CRC screening is not important or necessary [37,38,39]. The Medicare program needs to ensure that all Medicare beneficiaries are aware of the new policy, that part B does not require any deductible or coinsurance for screening colonoscopies. Eliminating the cost-sharing for therapeutic colonoscopies could be the next policy reform to be considered in further improving adherence to colorectal cancer screening [22].

Previous studies found divergent results of post-ACA changes in CRC screenings among the elderly and Medicare beneficiaries [22,24,26,34]. Some studies found an increase in the receipt of CRC screening [22,24], while others found no change in the use of any cancer screening procedure [26,34]. Unlike these studies, our study was able to use a longer time-frame to examine the effect of ACA policy changes on colonoscopy use. With this longer time-lapse since the implementation of the ACA, we found a significant effect of policy change, after controlling for many other potential factors affecting colonoscopy uptake. We even incorporated the availability of health care providers in the area as a control factor, something none of the earlier analyses had considered. The supply-side variable indicates that the availability of GIs in the geographic area affects the likelihood of receiving a colonoscopy within the recommended time frame. To achieve 80% coverage of colonoscopy, the availability of GIs in the country may become a significant limiting factor. Policy-makers and researchers should carefully evaluate the possibility of involving primary care providers in the provision of colonoscopies.

This study has several limitations. First, the BRFSS is based on self-reports, which may be subject to recall bias. The BRFSS did not carry out cross-checking of reported colonoscopy with individuals’ medical records [40,41]. Second, there is also a possibility of selection bias in this type of survey because less healthy patients may not be included in the sample. Third, the BRFSS does not include information about actual out-of-pocket expenditures or other non-medical expenses associated with the screening, such as opportunity cost associated with time or difficulty in scheduling colonoscopies [42,43]. Fourth, this study could not distinguish between screening and therapeutic colonoscopies and whether or not the ACA policy itself changed the providers’ behavior in terms of recommending screening or therapeutic colonoscopies. Fifth, any data comparing changes over the years is subject to the threat of validity, i.e., whether the change is due to policy shift or it indicates changes over time due to increasing awareness or changes in social norms in favor of CRC. Finally, colonoscopy coverage was defined as the receipt of colonoscopy in the previous ten years, the recommended frequency of colonoscopies. Clearly, some colonoscopies in the post-ACA period happened prior to the adoption of the ACA but since the same cut-off of 10-years was used pre-ACA and post-ACA, the policy shifter should be able to indicate the effect of the ACA, if any, on the coverage rate. The full effect of the ACA will be observable when the 10-year time frame falls within the post-ACA years, implying that the national data set of 2021 or beyond should be able to indicate the effect of ACA policy change on colonoscopy uptake. 

## 5. Conclusions

Following the implementation of the ACA, a statistically significant increase in colonoscopy use was observed. However, achieving the target rate of 80% coverage will require additional interventions to encourage higher levels of screenings.

## Figures and Tables

**Table 1:** Characteristics of Survey Participants Aged 65 to 75 Years: Behavioral Risk Factor Surveillance System (BRFSS), 2008–2016.

Variables	Total			Pre-ACA (2008, 2010)			Post-ACA (2012, 2014, 2016)		
	N	Weighted N	%	N	Weighted N	%	N	Weighted N	%
Total	349,899	20,760,005	100.0	125,577	7,292,561	100.0	224,322	13,467,444	100.0
**Age (years)**									
65–66	78,424	4,778,975	23.0	27,686	1,657,607	22.7	50,738	3,121,369	23.2
67–68	72,261	4,293,712	20.7	25,928	1,492,646	20.5	46,333	2,801,066	20.8
69–70	65,746	3,853,545	18.6	22,953	1,308,456	17.9	42,793	2,545,089	18.9
71–72	56,989	3,307,273	15.9	20,739	1,187,668	16.3	36,250	2,119,606	15.7
73–75	76,479	4,526,499	21.8	28,271	1,646,184	22.6	48,208	2,880,315	21.4
**Sex**									
Male	144,628	10,041,253	48.4	49,856	3,498,723	48.0	94,772	6,542,530	48.6
Female	205,271	10,718,752	51.6	75,721	3,793,838	52.0	129,550	6,924,915	51.4
**Race/ethnicity**									
Non-Hispanic White	300,176	16,335,132	78.7	107,573	5,787,662	79.4	192,603	10,547,470	78.3
Non-Hispanic Black	23,541	1,900,058	9.2	8262	625,413	8.6	15,279	1,274,645	9.5
Hispanic	11,393	1,464,786	7.1	4523	515,157	7.1	6870	949,629	7.1
Other	14,789	1,060,030	5.1	5219	364,328	5.0	9570	695,702	5.2
Married	191,812	13,062,607	62.9	67,832	4,791,316	65.7	123,980	8,271,291	61.4
**Region of Residence**									
Northeast	63,156	3,721,267	17.9	21,448	1,333,058	18.3	41,708	2,388,209	17.7
Midwest	83,992	4,610,164	22.2	27,263	1,611,787	22.1	56,729	2,998,377	22.3
South	117,730	7,807,516	37.6	45,098	2,740,775	37.6	72,632	5,066,741	37.6
West	85,021	4,621,059	22.3	31,768	1,606,941	22.0	53,253	3,014,117	22.4
**Household income**									
Less than $15,000	37,975	2,132,736	10.3	16,145	799,267	11.0	21,830	1,333,470	9.9
$15,000 to less than $25,000	71,719	3,999,888	19.3	28,776	1,493,125	20.5	42,943	2,506,764	18.6
$25,000 to less than $35,000	51,045	2,896,727	14.0	20,179	1,105,098	15.2	30,866	1,791,628	13.3
$35,000 to less than $50,000	61,885	3,648,907	17.6	22,442	1,296,287	17.8	39,443	2,352,620	17.5
$50,000 or more	127,275	8,081,747	38.9	38,035	2,598,784	35.6	89,240	5,482,963	40.7
**Education**									
Did not graduate high school	28,997	2,565,191	12.4	13,251	834,555	11.4	15,746	1,730,636	12.9
Graduated High School	107,851	6,286,535	30.3	43,068	2,350,414	32.2	64,783	3,936,121	29.2
Attended College	92,346	5,878,894	28.3	31,705	1,770,625	24.3	60,641	4,108,269	30.5
Graduated from College	120,705	6,029,384	29.0	37,553	2,336,966	32.0	83,152	3,692,418	27.4
**Colonoscopy within 10 years**									
No	113,624	6,815,019	32.8	46,619	2,660,497	36.5	67,005	4,154,522	30.8
Yes	236,275	13,944,986	67.2	78,958	4,632,063	63.5	157,317	9,312,922	69.2
Quartile of Gastroenterologist Availability *									
Q1	59,323	1,810,049	8.7	20,757	636,937	8.7	38,566	1,173,112	8.7
Q2	110,334	5,347,096	25.8	39,196	1,864,253	25.6	71,138	3,482,844	25.9
Q3	86,369	7,348,162	35.4	33,101	2,566,325	35.2	53,268	4,781,836	35.5
Q4	93,873	6,254,698	30.1	32,523	2,225,046	30.5	61,350	4,029,653	29.9
**Exercise in past 30 days**									
No	95,614	5,765,920	27.8	36,007	2,027,556	27.8	59,607	3,738,364	27.8
Yes	254,285	14,994,085	72.2	89,570	5,265,004	72.2	164,715	9,729,081	72.2
**Smoking Status**									
Current smoker	40,547	2,331,537	11.2	15,127	808,134	11.1	25,420	1,523,403	11.3
Former smoker	149,275	9,133,039	44.0	55,012	3,271,931	44.9	94,263	5,861,108	43.5
Never smoked	160,077	9,295,429	44.8	55,438	3,212,495	44.1	104,639	6,082,934	45.2

Note. * Gastroenterologist availability quartiles are determined by the number of gastroenterologists per 1000 (2010) in the respondent’s state. The American Medical Association (AMA) Health Workforce Mapper reports the availability of different specialists by state, and we have used the reported number of professionally active Gastroenterologists by the state to calculate the geographic availability of GIs per 1000 individuals in a given population. All analyses weighted by strata, primary sampling unit, and ranking-derived weights.

**Table 2:** Colonoscopy Use Before and After Implementation of The Affordable Care Act Policy Change: Behavioral Risk Factor Surveillance System (BRFSS), 2008–2016.

Variables	Colonoscopy Within the Past 10 Years		
	Pre ACA	Post ACA	Differences
	%	%	% Change
**Total**	63.5	69.2	8.9
**Age (years)**			
65–66	62.2	67.3	8.2
67–68	63.5	69.9	10.0
69–70	64.2	69.8	8.7
71–72	63.8	70.1	9.8
73–75	64.1	69.2	7.9
**Sex**			
Male	63.7	68.7	7.9
Female	63.4	69.6	9.8
**Race/ethnicity**			
Non-Hispanic White	65.5	70.9	8.2
Non-Hispanic Black	61.4	70.0	14.0
Hispanic	50.0	56.2	12.5
Other	54.8	59.2	8.1
**Married**			
Yes	66.7	72.4	8.6
No	57.5	64.0	11.4
**Region of Residence**			
Northeast	67.3	71.9	6.8
Midwest	64.7	70.2	8.4
South	63.9	70.2	9.8
West	58.5	64.2	9.8
**Household income**			
Less than $15,000	45.5	52.4	15.1
$15,000 to less than $25,000	55.7	61.1	9.6
$25,000 to less than $35,000	63.4	67.1	5.9
$35,000 to less than $50,000	66.4	71.0	6.9
$50,000 or more	72.1	76.8	6.5
**Education**			
Did not graduate high school	47.9	55.5	15.9
Graduated High School	61.2	66.8	9.3
Attended College	64.4	71.0	10.3
Graduated from College	70.8	76.0	7.3
**Quartile of Gastroenterologist Availability**			
Q1	59.5	66.5	11.8
Q2	64.2	70.4	9.6
Q3	61.2	66.9	9.3
Q4	66.7	71.5	7.2
**Exercise in past 30 days**			
No	57.0	63.0	10.5
Yes	66.0	71.5	8.3
**Smoking Status**			
Current smoker	49.0	55.7	13.8
Former smoker	66.8	71.8	7.6
Never smoked	63.8	69.9	9.5

**Table 3:** Multivariate Analysis of Factors Associated with Colonoscopy Use: Behavioral Risk Factor Surveillance System (BRFSS), 2008–2016.

Variables	Colonoscopy Within the Past 10 Years		
	AOR	(95% CI)	
**Policy shift**	1.15	1.08	1.22
**Age (years)**			
65–66	1.00		
67–68	1.11	1.06	1.16
69–70	1.15	1.09	1.20
71–72	1.16	1.11	1.22
73–75	1.16	1.11	1.21
**Sex**			
Male	1.00		
Female	1.19	1.15	1.22
**Race/ethnicity**			
Non-Hispanic White	1.00		
Non-Hispanic Black	1.16	1.10	1.23
Hispanic	0.78	0.72	0.84
Other	0.70	0.64	0.77
**Married**			
Yes	1.00		
No	0.84	0.82	0.87
**Region of Residence**			
Northeast	1.00		
Midwest	0.98	0.92	1.03
South	0.98	0.93	1.03
West	0.74	0.70	0.79
**Household income**			
Less than $15,000	1.00		
$15,000 to less than $25,000	1.25	1.18	1.33
$25,000 to less than $35,000	1.53	1.44	1.63
$35,000 to less than $50,000	1.69	1.59	1.80
$50,000 or more	2.10	1.97	2.24
**Education**			
Did not graduate high school	1.00		
Graduated High School	1.24	1.17	1.31
Attended College	1.37	1.29	1.45
Graduated from College	1.53	1.44	1.63
**Quartile of Gastroenterologist Availability**			
Q1	1.00		
Q2	1.15	1.11	1.19
Q3	1.06	1.02	1.10
Q4	1.15	1.10	1.21
**Exercise in past 30 days**			
No	1.00		
Yes	1.23	1.19	1.27
**Smoking Status**			
Current smoker	1.00		
Former smoker	1.71	1.63	1.80
Never smoked	1.50	1.43	1.57
**Years**	1.03	1.02	1.04

Note: All analyses weighted by strata, primary sampling unit, and ranking-derived weights. CI = confidence interval; AOR = Adjusted odds ratio.
